# Plausible Roles for RAGE in Conditions Exacerbated by Direct and Indirect (Secondhand) Smoke Exposure

**DOI:** 10.3390/ijms18030652

**Published:** 2017-03-17

**Authors:** Joshua B. Lewis, Kelsey M. Hirschi, Juan A. Arroyo, Benjamin T. Bikman, David L. Kooyman, Paul R. Reynolds

**Affiliations:** 1 Lung and Placenta Research Laboratory, Department of Physiology and Developmental Biology, Brigham Young University, Provo, UT 84602, USA; jblewis1101@gmail.com (J.B.L.); kelseyhirschi.6@gmail.com (K.M.H.); jarroyo@byu.edu (J.A.A.); 2 Laboratory of Obesity and Metabolism, Department of Physiology and Developmental Biology, Brigham Young University, Provo, UT 84602, USA; benjbnikman@byu.edu; 3 Laboratory of Osteoarthritis and Inflammatory Diseases, Department of Physiology and Developmental Biology, Brigham Young University, Provo, UT 84602, USA; davidlkooyman@byu.edu

**Keywords:** receptor for advanced glycation end-products (RAGE), secondhand smoke, disease, exposure

## Abstract

Approximately 1 billion people smoke worldwide, and the burden placed on society by primary and secondhand smokers is expected to increase. Smoking is the leading risk factor for myriad health complications stemming from diverse pathogenic programs. First- and second-hand cigarette smoke contains thousands of constituents, including several carcinogens and cytotoxic chemicals that orchestrate chronic inflammatory responses and destructive remodeling events. In the current review, we outline details related to compromised pulmonary and systemic conditions related to smoke exposure. Specifically, data are discussed relative to impaired lung physiology, cancer mechanisms, maternal-fetal complications, cardiometabolic, and joint disorders in the context of smoke exposure exacerbations. As a general unifying mechanism, the receptor for advanced glycation end-products (RAGE) and its signaling axis is increasingly considered central to smoke-related pathogenesis. RAGE is a multi-ligand cell surface receptor whose expression increases following cigarette smoke exposure. RAGE signaling participates in the underpinning of inflammatory mechanisms mediated by requisite cytokines, chemokines, and remodeling enzymes. Understanding the biological contributions of RAGE during cigarette smoke-induced inflammation may provide critically important insight into the pathology of lung disease and systemic complications that combine during the demise of those exposed.

## 1. Introduction

### 1.1. Global Burden

Currently, it is estimated that there are nearly 1 billion smokers worldwide (WHO Fact Sheet No 339). Of this enormous number, approximately 80% live in either low- or middle-income countries where the effects or burdens of tobacco-related illness and death are the most substantial. Furthermore, while the current worldwide population of smokers is estimated at 1 billion, current projections predict that this number will rise to 1.6 billion in the next twenty-five years [1]. With such an inordinate number of smokers world-wide, roughly 6 million people are expected to die each year because of tobacco exposure (WHO Fact Sheet No 339). Of this number, over 600,000 people will die prematurely as a result of exposure to secondhand smoke (SHS). Unfortunately, while these numbers themselves are galling, rampant tobacco use throughout the world has also had societal ramifications as exposure is believed to contribute to over $500 billion in damages annually [2].

Because it appears that smoking prevalence will continue to rise despite its inherent dangers and costs, research is expanding in order to better understand the consequences of these trends. The intent of this review is to highlight significant health outcomes that result from SHS exposure and suggest a generally unifying mechanistic theme underlying the biological consequences of exposure. Mounting evidence suggests that the signaling effects of receptors for advanced glycation end-products (RAGE) during exposure to primary and SHS may contribute to inflammatory disease establishment and progression. RAGE is expressed in a variety of cell types including endothelial and vascular smooth muscle cells, fibroblasts, macrophages/monocytes, osteoprogenitor cells, endothelium, and epithelium [3] (personal communication). Of note, RAGE is most abundantly expressed in the lung, the tissue in which it was initially discovered. Although RAGE is predominantly expressed in the lung, it is detectable in a variety of tissues including the heart, brain, placenta, liver, kidney, pancreas, small intestine, and colon [4,5]. This review will highlight the current understanding related to a subset of smoke-related pathologies and conclude with evidence that supports a role for RAGE in disease manifestation. The biochemical assessments performed to date have linked many tobacco-related substances with negative health consequences [6]; however, much remains to be discovered.

### 1.2. Tobacco Smoke

Tobacco smoke contains over 4000 chemical substances [6], and a large portion of these entities have been correlated with damaging health outcomes. The combustion of tobacco smoke produces numerous compounds observed in both gaseous and particulate fractions. Many of these compounds are toxic components that have been demonstrated to induce inflammation, cause irritation, asphyxiation, and even carcinogenesis. Recent studies have suggested that at least 45 of these substances are known carcinogens [7]. Some of the key toxins produced by tobacco smoke include benzene (leukemogen) [8], formaldehyde (an irritant and carcinogen) [9], benzo[*a*]pyrene (carcinogen) [10], carbon monoxide and cyanide (asphyxiants) [11], acrolein (an irritant) [12], and polonium (a radioactive carcinogen) [13,14]. Additionally, combustion of tobacco products creates a non-enzymatic reaction of reducing sugars and amino groups to create compounds known as advanced glycation end-products (AGEs) [15]. Such tobacco-derived AGEs are formed by Malliard chemical pathways and are the key ligand that perpetuates pro-inflammatory RAGE signaling [16]. AGEs that bind RAGE have been implicated in a large and diverse group of diseases including respiratory inflammatory diseases [17], cardiovascular disease [18], cancer [19], diabetes [20], neurodegenerative disorders [21], placental dysfunction [22,23], osteoarthritis [24], and general inflammation [25]. In mechanistic terms, AGE-RAGE interaction initiates a cascade of events that results in the induction of chronic inflammation and impaired cell survival [26,27].

While significant damage is induced by active smoking, research has demonstrated that individuals exposed to passive smoking (or secondhand smoke, SHS) are at risk for developing significant health problems [6,28]. Indeed, literature suggests that SHS may even expose individuals to higher levels of certain deleterious compounds than those observed in mainstream smoke. SHS for example is shown to have higher levels of PAHs [29,30], tobacco-specific nitrosamines (TSNA) [31,32,33], aromatic amines [34], aza-arenes [29,35], carbon monoxide [36,37,38], nicotine [39,40], ammonia [41], pyridine [42,43], and gas phase components of acrolein, benzene, toluene, and isoprene 1,3-butadiene [44]. Recently, thirdhand smoke has also been implicated as a potent source of exposure to the toxins found in cigarettes [45]. Thirdhand smoke is obtained when tobacco smoke constituents become deposited on surfaces and such deposits may undergo oxidation and other diverse chemical processes that result in the synthesis of carcinogenic species including TSNAs [46]. In fact, it has been speculated that the dangers associated with thirdhand smoke may be even more profound than active smoking [47] due to the process in which thirdhand smoke is generated. As these deposited substances are a product of time and isolation, thirdhand smoke poses real dangers for both active smokers and nonsmokers alike.

## 2. Health Outcomes and Comorbidities

### 2.1. Chronic Obstructive Pulmonary Disease

Chronic obstructive pulmonary disease (COPD) is one of the leading causes of mortality and morbidity and currently estimated to affect roughly 5% of the world’s population or about 329 million individuals (WHO, The top 10 Causes of Death Fact Sheet 2012). The data overwhelmingly implicate primary or active smoking as the greatest risk factor for developing COPD [48]; however, exposure to environmental tobacco smoke is also highly associated with increased risk for COPD in individuals who have never smoked [49]. Globally, COPD is projected to be the third leading cause of death by 2020. Economists have estimated that the economic burden (including both direct and indirect costs) resulting from COPD was $2.1 trillion in 2010, but believe that this will rise to $4.3 trillion by 2030 [50]. Direct costs alone have been estimated to be in the $49.9 billion range, suggesting a greater need for preventative measures, as well as improvements in earlier diagnosis and more cost effective treatment.

Without question, pharmacological and nonpharmacological interventions have contributed to improved outcomes as they relate to the management of COPD [51,52]. However, as the worldwide prevalence of COPD is predicted to increase, so will the urgency of improved comprehensive therapy. Inflammation intensifies as COPD progresses [53] and does not “burn out” as do many other chronic inflammatory diseases [54]. Therefore, there is a pressing need for the development of new molecular targets and associated therapies, particularly as no existing treatment has been shown to reduce disease progression. New therapies for COPD may arise from improvements in existing drugs (for example, longer acting β_2_-agonists and anticholinergics) or from the development of novel therapies when underlying disease processes are better understood. Despite recent advances in the understanding of COPD molecular pathogenesis [55], there is clearly a need for more research into its basic mechanisms. Even so, there are still several reasons why drug development in COPD has been difficult. Animal models of COPD for early drug testing are not very satisfactory [56,57]. Animal models have contributed important details related to understanding immune mechanisms; however, drugs aimed at ameliorating symptoms observed in animal models often fail in Phase II trials. One plausible explanation may be that mucus is not produced in the bronchial tree of mice, so mucous exacerbations in COPD cannot be modeled well [58]. Furthermore, human COPD is coincident with variable pathologies at different stages of COPD severity, whereas the three main current animal models merely imitate a subset of COPD characteristics following exposure to noxious stimuli, tracheal instillation of elastases, or genetic modifications.

The characterization of innovative drugs with promise is periodically met with uncertainty because of the culminating requirement of multi-year, long-term trials. Confounding trial design is the notion that COPD patients are often disqualified from participating due to notable comorbidities including diabetes and heart disease. Finally, new treatments are also slow to develop because there remains disagreement in the field as to optimal biomarkers in a patient’s sputum or blood necessary to evaluate acute amelioration and long-term therapeutic capacity. Despite these limitations, a lucid understanding of disease progression during primary and SHS exposure is essential in refining patient care.

In terms of the pathophysiology, COPD is typically characterized by airflow obstruction that is minimally reversible. This airflow obstruction is due to chronic inflammation and permanent pulmonary airspace enlargement as well as the loss of elastic recoil caused by apoptosis that destroys alveolar walls observed in emphysema. Persistent inflammation in COPD patients is characteristic not only in the airways, but in the respiratory parenchyma and pulmonary vasculature as well, and results in disruption of normal lung function specifically through remodeling of the distal pulmonary airspaces. Because chronic inflammation is a major defining characteristic of the disease, extensive research surrounding pro-inflammatory molecular mechanisms have been conducted. The key aim of such research focuses on the attenuation or removal of chronic inflammation that overcomes natural protective mechanisms and the resulting tissue damage seen with COPD. Contributors to this inflammation-related process include imbalances between proteases/antiproteases, oxidative stress, elevated apoptotic indexes, and enhanced neutrophil, macrophage, and T lymphocyte extravasation.

Recent reports have corroborated previous findings that neutrophils are increased in sputum of patients with COPD along with increased interleukin-6 (IL-6) signaling [59]. Substantial evidence has implicated primary or active smoking as a major contributor to the recruitment of these neutrophils; however, mounting evidence now suggests that secondhand smoke may have a similar effect on neutrophils [60] that is likely mediated through similar interleukin signaling [61,62]. Under normal physiological conditions, neutrophils employ proteases and small cationic peptides to attack invading bacteria, viruses, and harmful exogenous material such as particulates found in tobacco smoke. Yet, in chronic inflammatory conditions, neutrophils become major destructors of the alveolar elastic matrix. These neutrophils also release enzymes and other mediators that cleave collagen into fragments that may further activate inflammatory cells [63]. One potent signaling factor that has been demonstrated to drive neutrophilic infiltration is the chemoattractant interleukin-8 (IL-8) which is produced by exposed and damaged epithelium and endothelium [64,65]. Additionally, other chemoattractants that have been shown to induce neutrophil migration include chemokine CXC motif ligands 1, 2, 5, 8 (CXCL-1, 2, 5, 8) [66,67,68], leukotriene B4 (LTB4) [68], IFN-ϒ [69], IL-1β [70,71], and TNF-α [72]. Current data increasingly suggest that these potent inflammatory chemoattractants are elevated with exposure to SHS [73,74]. While neutrophils are predominant mediators of chronic inflammation, they are not the only important pro-inflammatory mediator. Macrophages have also been shown to participate in the propagation of inflammation through the release of chemoattractants, and are elevated in the airways, parenchymal bronchoalveolar lavage fluid (BALF), and sputum [75,76,77] from affected patients. Studies involving the exposure of mice to SHS have demonstrated increased macrophages in response to SHS [78]. Furthermore, as these adaptive immunity cells play such a vital role in chemoattraction, it is unsurprising that research suggests macrophage recruitment closely corresponds with the severity of the disease [79]. Like neutrophils, macrophages migrate to injured lung tissue and enhance the release of TNF-α, IL-8, CXC chemokines, monocyte chemotactic peptide-1 (MCP-1), LTB4, and other pro-inflammatory molecules [55]. Finally, it should be noted that research indicates that T-cells may act as important intermediaries in the development of emphysema [80]. In a comparison of normal patients and those with smoke-induced COPD, diseased patients demonstrated elevated levels of CD3 and CD8 [80], two cytotoxic t-cell subgroups that organize apoptotic pathways used to kill infected or damaged cells. CD8 in particular was shown to be highly correlated with increasing severity in emphysema patients [80]. A recent analysis of mice subjected to SHS resulted in increased levels of CD4 and CD8; conversely, inhibition of these cells prevented airspace enlargement, inhibited cytokine release, and reduced apoptotic signaling [81]. Mechanistically, it is likely that CD8 subtly interacts in conjunction with CD4, a T-helper cell whose activation releases cytokines and helps orchestrate the migration and activity of other inflammatory cells. These T-cell mediated processes seemingly disrupt autoimmune regulation, thus enhancing perpetual inflammation.

### 2.2. Cancer

It is estimated that cigarette smoking contributes to 30% of all cancer deaths in developed countries [82]. Tobacco smoke is believed to be responsible for 70% of lung cancers deaths [83] (approximately 1.3 million deaths each year [84]) and 42% of esophageal and oral cavity cancer deaths [85]. Furthermore, tobacco smoke is believed to contribute significantly to the development of cancers of the larynx, urinary bladder, and pancreas, and to a lesser extent to cancers of the kidney, stomach, cervix, and myeloid leukemia [86]. Current evidence largely implicates active smoking as a major risk factor in cancer development; however, mounting evidence now suggests that SHS may equally participate. SHS has been shown to increase the risk of developing lung [87], oropharyngeal [88], colorectal [89], breast [88], cervical [90], bladder [90], and pancreatic cancer [91]. Moreover, studies investigating nitrosamines, some of the most potent carcinogens in tobacco smoke, have demonstrated that high levels are present in both mainstream and SHS [92]. As nitrosamines are readily absorbed through the alveoli and then rapidly distributed through the blood, it is unsurprising that they are found to play a major role in the induction of many cancers [93,94].

Compounds such as PAHs, aromatic amines, aza-arenes, carbon monoxide, TSNA, nicotine, ammonia, pyridine, and the gas compounds of acrolein, toluene, isopentene-1,3-butadiene, and benzene are common in SHS. Overwhelmingly, the data implicate these substances in a host of cancers, although the mechanisms by which this takes place are broad and diverse. For example, recent data have demonstrated that benzene [95], toluene [95], and nicotine [96] have the ability induce the upregulation of CYP2E1, an enzyme that activates many foreign chemical compounds to become ultimate toxicants [97]. Aside from the ability to produce toxicants, the induction of CYP2E1 has been suggested to be the first step in leading to chemically induced carcinogenesis [98]. Alternatively, PAHs and TSNA has been shown to increase epithelial to mesenchymal transition (EMT), which is closely associated with an invasive or metastatic phenotype. Increased EMT is characterized by a downregulation of genes encoding for epithelial junction (claudins, occludins, e-cadherin) as well as an activation of protein products that promote mesenchymal adhesion. As these epithelial junctions are crucial in regulating cell differentiation, proliferation, and polarity, it is unsurprising that the loss of these proteins is often associated with an invasive phenotype [99]. As these tissues transition from an epithelial to mesenchymal state, the epithelial barrier is disrupted and thus a key initial line of defense in the innate immune system is compromised.

In general, tobacco smoke seems to broadly influence carcinogenesis in four ways. First, the gas and particulate phase of tobacco smoke includes at least 20 substances that can induce lung tumors in rodents [100,101]. These compounds directly contribute to carcinogenesis. Second, tobacco smoke includes substances that are not directly carcinogenic alone, but enhance the activity of carcinogens when co-administered. These substances include tumor promoters, co-carcinogens, and toxicants such as catechol, methyl catechols, and PAHs [102]. One potent example of such a compound is acrolein, which is not strongly carcinogenic when in isolation. However, acrolein expressed by ciliated epithelium can be highly toxic due to the hindrance of clearing tobacco smoke compounds from the lung, resulting in profound exposure to other carcinogens. Furthermore, acrolein reacts directly with DNA and protein, thus triggering genomic silencing of gene targets [103] that may enhance the likelihood of carcinogenesis. Third, tobacco smoke substantially influences the chronic inflammatory microenvironment. Tobacco smoke causes the recruitment of inflammatory cells, cytokine and chemokines that can act as drivers for cancer development and progression [104]. It is well documented that the infiltration of tumor-associated macrophages in tumor lesions is common to a host of cancer types, and is associated with tumor angiogenesis, invasion, and metastasis [105,106,107,108]. Finally, matrix metalloproteinases (MMP)-1, MMP-8, MMP-9, and MMP-13 are collagenases implicated in the development of COPD in response to cigarette smoke [109]. Interestingly, these same MMPs with notable importance in emphysema also function in tumor invasion and metastasis [110]. In fact, recent data supports the notion that elevated collagenases is associated with an increase in the severity of cancer. Because MMPs degrade the ECM in a fashion that allows tumor cells to be released from binding factors in their environment, greater MMP abundance increases the motility of cancer cells [111].

### 2.3. Developmental Complications

As cigarettes are known to be one of the most common teratogens [112], a number of serious obstetric complications arise with cigarette smoke exposure during pregnancy [113]. Approximately 10% of pregnant women in the US smoke, thereby exposing nearly 400,000 fetuses yearly to tobacco specific toxins [114]. Exposure to smoke during pregnancy has been demonstrated to increase the likelihood of congenital limb deficiencies [115], congenital heart defects [116], orofacial clefting [112], and many other developmental abnormalities. Active smoking has long been considered a teratogenic agent that increases the risk of premature birth, but recent data shows that 22%–30% of nonsmoking pregnant women exposed to SHS are also at risk [117]. Developmental defects in the fetus represent substantial pregnancy complications; however, perinatally, smoke exposure further enhances mortality via increased risk of sudden infant death syndrome (SIDS) and preterm birth [118]. Altogether, it not surprising that many researchers have suggested that cigarette exposure may be the single most important avoidable cause of adverse pregnancy outcomes [119,120,121].

Nicotine, one of the primary addictive compounds in tobacco smoke, is a key substance that contributes significantly to many of these health problems as even minute levels induce detectable transcriptomic modifications in small-airway epithelium [122]. Nicotine readily crosses the placenta [123] and binds to nicotinic acetylcholine receptors (nAChRs) which regulate fetal brain development [124]. Interestingly, research has demonstrated that nicotine levels are higher in the amniotic fluid, fetal serum, and placenta than in the corresponding maternal serum [125]. Studies demonstrating the adverse effects of tobacco smoke on neurodevelopment have provided compelling evidence that nicotine increases cellular damage, reduces overall cell number, impairs synaptic activity, and influences processes such as cell replication to differentiation and apoptosis [126,127,128]. Furthermore, nicotine has been associated with adverse neurocognitive outcomes such as behavioral disorders [129], cognitive dysfunction [130], and attention deficit hyperactivity disorder [131,132].

While a significant portion of the literature implicates tobacco smoke in neurodevelopmental pathologies, such effects are not limited to the nervous system. Prenatal tobacco smoke exposure has been demonstrated to have striking effects on respiratory development in that it reduces respiratory compliance in infants and impairs lung function in school-aged children [114,133]. Possibly contributing to impaired lung function are data that suggest that maternal smoke exposure may alter Clara cell secretory protein (CCSP) expression in fetal lungs [134]. Indeed, evidence currently suggests that maternal smoke exposure (including SHS) during pregnancy leads to the deregulation of gene expression [135]. Confirmatory primate studies have shown that in utero nicotine exposure adversely affects overall lung development by decreasing lung size and volume, elastin, while increasing Type I and Type III collagen, alveolar volume, and airway wall areas [136,137,138,139]. While the immediate ramifications are apparent, researchers have shown that nicotine exposure not only predisposes the fetus to lung dysfunction, but also has the ability to influence asthma in second and third generation offspring, likely through epigenetic modulation of the fetal program [140,141,142]. Aside from respiratory disorders, nicotine has further been shown to affect endocrine function [143,144], increase the likelihood of the fetus to develop chronic kidney disease (CKD) through increased mitochondrial dysfunction [145], and decrease auditory response and auditory development [146,147,148]. Overwhelming, the data suggests a particularly insidious role for SHS and its ability to influence development.

One further factor that may have a causal role in many developmental deficiencies is the impact of premature delivery, a risk factor that tobacco smoke has been shown to significantly increase [149]. As tobacco smoke has been shown to increase preterm birth (PTB), it should be noted that it additionally exacerbates intrauterine growth restriction (IUGR) and preeclampsia (PE), two placental diseases closely associated with PTB [113,150,151]. IUGR is a complication that stems primarily from uteroplacental vascular insufficiency, which ultimately creates an environment of chronic oxygen and nutrient deficiency, resulting in restricted fetal growth [152]. PE is another disease that impacts placentation wherein maternal hypertension and proteinuria accounts for around 20% of induced PTB [153]. Because complications such as perinatal hypoxia and asphyxia, cerebral palsy, and persistent pulmonary hypertension of the newborn have been associated with both IUGR and SHS exposure [154,155], it is likely that SHS modulates IUGR and PE symptoms that may culminate in diverse developmental pathologies

### 2.4. Cardiometabolic Disorders

The intimate connection, both in etiology and outcome, of cardiovascular and metabolic processes has resulted in the term, “cardiometabolic diseases”. The relevance of this is highlighted in the numbers: heart disease is the leading cause of death [156] and insulin resistance is the most common disorder in the US, affecting half of all adults [157]. Because of these startling statistics, considerable effort has been devoted over recent decades to elucidate effective strategies to reverse the trends. Overwhelmingly, these efforts have focused on the role of lifestyle variables, particularly diet. However, while diet is clearly relevant [156,158], it is also clearly not the entire solution, as cardiometabolic diseases continue unabated. Indeed, such a paradigm has left relatively unexplored that what we inhale may matter as much as what we ingest.

Insulin resistance is the “metabolic” in cardiometabolic disorders. Due to the obvious challenges of determining causality of a cigarette smoke-insulin resistance interaction, most of the findings in humans are correlational in nature [159,160], though limited data exist to establish [161,162] that cigarette smoke exposure increases insulin resistance. Typified by a reduced ability of insulin to elicit action at cells throughout the body, as well as general hyperinsulinemia, insulin resistance is at the heart of most cardiometabolic disorders, such as hypertension [163,164], atherosclerosis [163], dyslipidemia [165], cardiomyopathy [166], and more [167,168].

Unsurprisingly, cigarette smoke exposure similarly increases the risk of myriad cardiovascular complications through diverse mechanisms, though insulin resistance is clearly a dominant factor [169]. For example, dyslipidemia (i.e., increased triglycerides, reduced high density lipoprotein cholesterol), which is a key predictor in cardiovascular mortality with cigarette smoking [170], is significantly worse in smokers with insulin resistance compared with more insulin-sensitive smokers [171,172].

A second instance of the role of insulin resistance in smoke-induced cardiometabolic disorders is abnormal endothelial physiology. Blood vessels from smoke-exposed humans are less dynamic, having a reduced dilatory capacity [173], and have increased leukocyte adherence [174], increasing the risk of clot formation. In regards to endothelium-dependent vasodilation, current evidence shockingly revealed that after only 15 to 30 min of breathing SHS, vasodilation of coronary arteries in non-smokers was impaired almost to the extent of habitual smokers [175]. Intriguingly, both of these pathological processes are associated with endothelial dysfunction and are exacerbated by insulin resistance [176,177].

Among the multiple mechanisms that mediate smoke-induced cardiometabolic disorders, the effects of smoke on lipid metabolism are noteworthy insofar as they may reveal a strategy to partially mitigate the cardiometabolic consequences of smoke exposure. In particular, cigarette smoke pathologically alters sphingolipid metabolism, resulting in the accrual of ceramides, the backbone of higher-order sphingolipids, in heart [162] and skeletal muscle [178]—two key insulin-responsive tissues. Ceramide accrual in these tissues resulted in substantial disruption of mitochondrial function, including alterations in morphology and electron transport. Moreover, smoke exposure altered insulin signaling in skeletal muscle. An increase in the action of serine palmitoyltransferase, the rate-limiting step in sphingolipid biosynthesis, was necessary for the ceramide accrual, as inhibition via myriocin, was protective against the deleterious effects of smoke exposure.

Data collected over the past few decades suggest that SHS increases the incidence of coronary heart disease approximately 25%–30% [179,180,181]. Furthermore, although active smokers receive up to 100 times the dose of smoke than individuals exposed to SHS, an active smoker’s relative risk of coronary heart disease is 1.78 followed closely by a passive smoker at 1.31 [182]. SHS contributes to cardiovascular disease by activating blood platelets [183] likely through the combined elevation of both fibrinogen [182,184] and thromboxane [185], thus leading to the development of artherosclerosis. SHS has also been demonstrated to decrease levels of NO, the primary substrate that is implicit in the hemodynamic changes in the vascular system [186]. Research has even demonstrated that, after only 20 min of SHS exposure, direct endothelial cell injury is observed. Mechanistically, SHS exposure has been shown to increase free radicals [187] while decreasing antioxidants [188], decrease mitochondrial function [178], decrease protective HDL levels [189,190], and increase arterial stiffness [191]. Such staggering data adds new meaning to the current warnings from the Surgeon General that state “there is no safe level of exposure to tobacco smoke” [114].

### 2.5. Joint and Movement Disorders

Osteoarthritis (OA), characterized by joint pain, effusion, loss of mobility, and deformity that progresses to functional joint failure, is one of the most common chronic diseases. It is reported to be the most common disease associated with the temporomandibular joint (TMJ) [192]. There is not currently any treatment to slow or stop the progression of OA. It has become the most common cause of long-term disability, in large part because of its association with the knee and spine. The incidence of OA in the population is comparable to other major disorders such as end-stage kidney disease and heart failure. For instance, there are nearly half a million joint replacements performed annually in the United States alone [193]. Many studies, including mouse knee destabilization and TMJ misalignment models, have demonstrated a pattern of biomarker expression associated with the progression of OA [194,195,196,197]. The disease appears to be associated with an initial rise in Tgf-β1 expression, followed by the upregulation of HtrA1, Ddr2 and Mmp13 expression, resulting in OA as assessed by standardized joint scoring methods such as the Mankin and the Osteoarthritis Research Society International (OARSI) scoring systems [198,199,200,201]. Curiously, the expression of HtrA1 and the other factors associated with OA are attenuated in a receptor for advanced glycation end-products (RAGE) knockout (KO) mice following surgically induced OA models [195]. This suggests that inflammation may be the trigger for the initiation and onset of OA. It follows that OA is associated with cigarette smoke. It is noted that, early on, the interaction of smoking and OA was controversial [202,203,204,205,206]. However, it has been reported that the discrepancies between smoking and OA interaction are likely do to study design and metrics [203,207]. A correlation between smoking and OA and/or cartilage defects is now apparent [207,208]. It is interesting to note that one study showed that the harmful effects of smoking associated with OA were due to both cartilage loss as well as the development of cartilage defects in people with a family history of joint disease [207]. Suggesting that a pre-disposition may be exacerbated by smoking through some bone/cartilage development association. Finally, it is noteworthy that one group who reported no association between direct smoking and OA did report a correlation between the joint disease and indirect smoking [206]. It is unknown if constituents of tobacco smoke have direct deleterious effects on chondrocyte function or if direct and/or indirect cigarette smoke induces cartilage damage through more global means such as inflammation.

## 3. RAGE: A Plausible Unifying Mechanism

Although many interrelated mechanistic processes potentially contribute to the diversity of diseases stemming from exposure to primary smoking, SHS, and thirdhand smoke, RAGE signaling is a program that commonly emerges. An underlying mechanistic theme of the smoke-related disease states outlined in this review is chronic inflammation, in which RAGE is a key modulator. Essential to understanding the clear link between RAGE and disease progression is the key concept that RAGE expression is increased by exposure to tobacco smoke [5,209,210,211,212] and the induction of RAGE causes inflammatory disease symptoms similar or identical to the ones described herein [23,195,213,214,215,216,217].

RAGE is expressed in a variety of cell types including endothelial and vascular smooth muscle cells, fibroblasts, macrophages/monocytes, osteoprogenitor cells, endothelium, and epithelium [3] (personal communication). Of note, RAGE is most abundantly expressed in the lung, the tissue in which it was initially discovered. Although RAGE is predominantly expressed in the lung, it is detectable in a variety of tissues including the heart, brain, placenta, liver, kidney, pancreas, small intestine, and colon [4,5]. RAGE is a pattern recognition cell surface receptor that binds many endogenous and exogenous entities such as S100/calgranulins [218], amyloid-β-peptide [219], HMGB-1 [220], and AGEs [221]. Following RAGE-ligand interaction, a cascade of signaling events elicits gene expression modulation via divergent signal transduction pathways [222,223,224]. Because RAGE expression can also increase when ligands accumulate [225], RAGE-ligand interactions may not only induce the defects described in this review, but also contribute to the chronicity of inflammatory tobacco smoke exposure observed in these pathological states as well. RAGE activation exacerbates a host of pro-inflammatory responses via MAP kinases (ERK, JNK, and P38), NF-κB, reactive oxidative species (ROS), and other chemokine mediators including TNF-α, IL1-β, and others [226]. While redundancies exist within the pathway, RAGE signaling generally culminates in the activation of NF-κB, a transcriptional regulator that not only promotes pro-inflammatory mediator elaboration, but also de novo RAGE expression. Thus, RAGE signaling via NF-κB represents a vicious positive feedback loop that orchestrates chronic inflammation. In contrast to short-lived cellular activation mediated by lipopolysaccharide (LPS), engagement of RAGE by its ligands results in prolonged inflammation [227] that, if left unchecked, causes severe tissue injury.
